# Synaptic Tau Seeding Precedes Tau Pathology in Human Alzheimer's Disease Brain

**DOI:** 10.3389/fnins.2018.00267

**Published:** 2018-04-24

**Authors:** Sarah L. DeVos, Bianca T. Corjuc, Derek H. Oakley, Chloe K. Nobuhara, Riley N. Bannon, Alison Chase, Caitlin Commins, Jose A. Gonzalez, Patrick M. Dooley, Matthew P. Frosch, Bradley T. Hyman

**Affiliations:** ^1^Department of Neurology, Harvard Medical School, MassGeneral Institute for Neurodegenerative Disease, Massachusetts General Hospital, Charlestown, MA, United States; ^2^C.S. Kubik Laboratory for Neuropathology, Harvard Medical School, Massachusetts General Hospital, Boston, MA, United States

**Keywords:** tau, seeding, propagation, synapses, aggregation, Alzheimer's disease

## Abstract

Alzheimer's disease (AD) is defined by the presence of intraneuronal neurofibrillary tangles (NFTs) composed of hyperphosphorylated tau aggregates as well as extracellular amyloid-beta plaques. The presence and spread of tau pathology through the brain is classified by Braak stages and thought to correlate with the progression of AD. Several *in vitro* and *in vivo* studies have examined the ability of tau pathology to move from one neuron to the next, suggesting a “prion-like” spread of tau aggregates may be an underlying cause of Braak tau staging in AD. Using the HEK293 Tau^RD^-P301S-CFP/YFP expressing biosensor cells as a highly sensitive and specific tool to identify the presence of seed competent aggregated tau in brain lysate—i.e., tau aggregates that are capable of recruiting and misfolding monomeric tau—, we detected substantial tau seeding levels in the entorhinal cortex from human cases with only very rare NFTs, suggesting that soluble tau aggregates can exist prior to the development of overt tau pathology. We next looked at tau seeding levels in human brains of varying Braak stages along six regions of the Braak Tau Pathway. Tau seeding levels were detected not only in the brain regions impacted by pathology, but also in the subsequent non-pathology containing region along the Braak pathway. These data imply that pathogenic tau aggregates precede overt tau pathology in a manner that is consistent with transneuronal spread of tau aggregates. We then detected tau seeding in frontal white matter tracts and the optic nerve, two brain regions comprised of axons that contain little to no neuronal cell bodies, implying that tau aggregates can indeed traverse along axons. Finally, we isolated cytosolic and synaptosome fractions along the Braak Tau Pathway from brains of varying Braak stages. Phosphorylated and seed competent tau was significantly enriched in the synaptic fraction of brain regions that did not have extensive cellular tau pathology, further suggesting that aggregated tau seeds move through the human brain along synaptically connected neurons. Together, these data provide further evidence that the spread of tau aggregates through the human brain along synaptically connected networks results in the pathogenesis of human Alzheimer's disease.

## Introduction

Primary tauopathies encompass a major class of neurodegeneration that is defined by those neurological disorders containing intracellular accumulations of the microtubule-associated protein tau. Of the primary tauopathies that exist, Alzheimer's disease (AD) is the most common. In addition to intraneuronal neurofibrillary tangles (NFTs) composed of hyperphosphorylated tau aggregates, extracellular plaques that result from the accumulation of the amyloid-beta peptide are a prominent pathology in the AD brain (Hyman et al., 1984; Braak and Braak, 1991). While both of these pathological hallmarks are required for the classification of AD, there appears to be a more direct correlation between the accumulation of tau pathology and cognitive decline (Arriagada et al., 1992; Zhou et al., 2012; Xia et al., 2017).

One of the major contributions to the idea of tau pathology spreading through the brain in a “prion-like” manner is the observation made by Braak and Braak, whereby tau pathology manifests in a consistent spatiotemporal pattern in human AD brains (Braak and Braak, 1991). It has been hypothesized this pattern suggests a transneuronal propagation of tau aggregates from one brain region to the next. *In vitro* studies have observed the ability of tau aggregates to move along axons and dendrites in neuronal cultures, both antero- and retrograde, as well as the ability of tau aggregates to leave one neuron and be taken up by another (Wu et al., 2013; Calafate et al., 2015; Takeda et al., 2015). *In vitro* studies have further demonstrated the ability of tau aggregates to behave similar to prions in that they possess the ability to recruit and ultimately template the naïve monomeric version of the protein. Building on this *in vitro* work, *in vivo* models have been generated to better study this “prion-like” propagation of tau pathology along synaptically connected networks in the intact brain. Aggregates of tau injected into mouse brains appear to move from the site of injection to synaptically connected regions (Clavaguera et al., 2013; Iba et al., 2013; Sanders et al., 2014; Calafate et al., 2015; Kaufman et al., 2017; Narasimhan et al., 2017). Further, when human tau is expressed selectively in the entorhinal cortex, it has been shown to propagate to the adjoining dentate gyrus of the hippocampus, suggesting that tau pathology is capable of traversing neural networks in the living brain (de Calignon et al., 2012; Liu et al., 2012).

This phenomenon of tau propagation in the human brain has thus far been based almost exclusively on cross-sectional staining for tau pathology in post-mortem tissue at different points in AD disease progression. As tau imaging markers have evolved, additional studies have been performed looking at the deposition of tau pathology in association with brain atrophy using positron emission tomography (PET) in living patients, finding that the formation of tau pathology—as defined by tau PET ligand binding—occurs along the immunohistologically described Braak Tau Pathway and directly correlates with neuronal dysfunction and ultimately loss (Ishiki et al., 2015; Ossenkoppele et al., 2016; Schöll et al., 2016; Sepulcre et al., 2016; Hoenig et al., 2018; Iaccarino et al., 2018). These studies demonstrate that NFTs systematically form first in layer 2 of the entorhinal cortex and, from there, progress to the hippocampal formation, posterior parahippocampal gyrus, anterior cingulate cortex, visual association area, and ultimately culminate in the primary visual cortex. Similar to what has been observed in tau transgenic mice (Holmes et al., 2014), tau “seeding”—the ability of tau aggregates to recruit and misfold naïve monomeric tau—can be detected prior to the development of overt tau pathology in human AD brains (Furman et al., 2017). We sought to expand on these results and test tau seeding levels along the Braak Tau Pathway. In addition to measuring tau seeding in cortical gray matter regions where neuronal cell bodies and NFTs are abundant, we examined tau seeding in (1) the axons by looking at the frontal white matter tracts and optic nerve and (2) the synapses by isolating synaptosomes from different regions along the Braak Tau Pathway in an effort to determine if aggregated tau can traverse neural networks through transneuronal propagation in the human AD brain. Together, these experiments add to the growing body of evidence that tau aggregates capable of corrupting naïve tau can move through the human brain along synaptically connected neuronal networks, resulting in the progression of AD.

## Materials and methods

### Human post-mortem brain collection

Human tissue samples were obtained from the Massachusetts Alzheimer's Disease Research Center Neuropathology Core at the MassGeneral Institute for Neurodegenerative Disease (Table 1). All brains were initially sliced coronally at the time of autopsy and immediately flash frozen between metal plates on dry ice. All brain sections were stored at −80°C. At the time of collection, approximately 1-cm of the desired brain region was dissected out of the frozen brain section and kept at −80°C until homogenization. To test for tau seeding along the Braak Tau Pathway, the following brain regions were collected under the guidance of a MGH neuropathologist from 29 human patients ranging from Braak I—Braak VI: Entorhinal cortex, Hippocampus, Posterior Parahippocampal Gyrus, Anterior Cingulate, Visual Association Area, Primary Visual Cortex, and Cerebellar Vermis. All cases had been formerly examined by an MGH neuropathologist to generate a neuropathological diagnosis. Additionally, all cases were blindly assessed for (1) the density of neuritic beta amyloid plaques based on a scoring system developed by the Consortium to Establish a Registry for Alzheimer's disease (Morris et al., 1989), (2) the distribution of beta amyloid plaques (Thal score) (Thal et al., 2002), and (3) the Braak tau stage based on the location of neurofibrillary tau tangles as determined by a total tau immunostaining and Bielchowsky's silver stain (Braak and Braak, 1991). These three scores were used to generate an Alzheimer's disease neuropathologic change (ADNC) rating or “ABC score” (Hyman et al., 2012).

**Table 1:** Human demographics and neuropathological data.

**Case #**	**Age at death**	**PMI**	**NPDX**	**Braak**	**ADNC**			**CERAD**	**Figure**
					**A**	**B**	**C**		
2064	50–55	14	FTD	0	0	0	0	0	4
2065	66–70	49	FTD	0	0	0	0	0	4
2067	60–65	46	ALS	0	0	0	0	0	4
2075	46–50	38	ALS	0	0	0	0	0	4
1841	66–70	12	Superficial Siderosis	I	1	1	1	A possible	3
1893	80–85	14	CVD	I	2	1	1	A possible	3
1928	>90	18	CVD	I	0	1	n/a	0	1, 3, 4
1965	76–80	48	Control	I	1	1	1	A possible	3, 5
2038	76–80	6	Control	I	0	1	0	0	4
2041	70–75	24	PD	I	0	n/a	n/a	n/a	4
1872	>90	22	CVD	II	3	1	1	A possible	1, 3, 4
1504	75–80	12	DLB	II	n/a	2	1	A possible	3
1755	80–85	6	PD	II	n/a	1	n/a	n/a	3, 5
1777	86–90	24	AD	II	0	1	0	0	3, 5
1900	86–90	16	LBD	II	3	1	2	B probable	3
1972	66–70	23	PD	II	2	1	1	A possible	3, 5
2068	76–80	9	Control	II	0	0	1	0	4
1759	60–65	12	DLB, AD	III	2	2	2	B probable	3
1854	80–85	6	AD	III	1	2	1	A possible	3, 5
1876	>90	18	AD	III	3	2	2	B probable	3
1899	86–90	14	AD	III	3	n/a	n/a	B probable	3
1907	86–90	14	AD	III	1	n/a	n/a	A possible	3, 5
1946	86–90	14	Control	III	0	2	n/a	0	3
1968	86-90	10	AD	III	3	2	2	B probable	3, 5
1516	>90	16	AD	IV	n/a	1	1	A possible	3
1820	80–85	20	AD	IV	3	3	3	C definite	3
1827	86–90	5	AD	IV	3	3	2	B probable	3
1970	86–90	16	AD	IV	2	3	2	B probable	3
2040	>90	23	AD	IV	3	2	1	A possible	4
1906	70–75	12	AD	V	3	2	2	B probable	3
1926	80–85	6	AD	V	3	3	2	B probable	3
1948	66–70	18	AD	V	n/a	n/a	n/a	B probable	3, 5
1963	76–80	15	AD	V	3	3	2	B probable	3, 5
1971	76–80	22	AD	V	3	3	2	B probable	3
1987	76–80	7	AD	V	3	3	3	C definite	3, 5
2019	80–85	12	AD	V	3	3	3	C definite	4
2021	80–85	24	AD	V	3	3	2	B probable	4
1810	76–80	7	AD	VI	3	3	3	C definite	3, S2
1845	>90	19	AD	VI	3	3	3	C definite	3, 5
2023	80–85	n/a	AD	VI	3	3	3	C definite	1, 4, S2
2026	>90	12	AD	VI	3	3	3	C definite	4
2037	70–75	22	AD	VI	3	3	3	C definite	4
2039	70–75	24	AD	VI	3	3	3	C definite	4
2056	>90	9	AD	VI	3	3	3	C definite	1, 4, S2
2057	80–85	39	AD	VI	3	3	2	B probable	4
2066	86–90	24	AD	VI	3	3	3	C definite	1, 4, S2
2069	86–90	12	AD	VI	3	3	3	C definite	1, 4, S2

*AD, Alzheimer's Disease; PD, Parkinson's Disease; FTD, Frontotemporal Dementia; ALS, Amyotrophic Lateral Sclerosis; DLB, Dementia with Lewy Body Disease; CVD, Cerebrovascular Disease; ADNC, Alzheimer's Disease Neurological Changes; NPDX, Neuropathologic diagnosis; PMI, Post-mortem interval*.

### Immunohistochemistry

The contralateral hemisphere of all cases that had sections obtained for biochemical analysis was post-fixed in formalin. Brain regions of interest were paraffin-embedded and cut into 8 μm thick tissue sections and subsequently mounted on slides. All sections were de-paraffinized with xylene followed by a descending ethanol series. Slides were incubated in 3% H_2_O_2_ immediately prior to immunolabeling. For the Aβ stain, sections were first incubated with 90% formic acid for 5 min. All sections were then rinsed with TBS and incubated with either 1:3,000 rabbit polyclonal anti-tau (DAKO) or 1:200 mouse monoclonal anti-amyloid beta (Clone 6F/3D, DAKO) for 1 h at room temperature. Immunolabeling was then visualized using Vectastain rabbit or mouse IgG horse-radish peroxidase (HRP) kits (VectorLabs) and Vector peroxidase substrate kit DAB (VectorLabs). Slides were then dehydrated in ascending ethanol/xylene and coverslipped with Cytoseal XL. For the Bielschowsky silver stain, sections were prepared the same as above and de-paraffinized with xylene followed by a descending ethanol series. Slides were incubated with 20% silver nitrate (diluted in distilled water) for 15 min in the dark, silver nitrate solution set aside, and slides washed once with distilled water. The saved silver nitrate was titrated with concentrated ammonium hydroxide until the precipitate disappeared and slides then incubated with this ammoniated silver nitrate solution in the dark for 10 min. The ammoniated silver nitrate solution was removed and set aside. Slides were washed in ammonia water (1 mL concentrated ammonium hydroxide in 1 L distilled water) for 1 min followed by 1 min in distilled water. 0.5 mL developer solution (20 mL 10% formalin, 0.5 g citric acid, 100 mL distilled water, 1 drop concentrated nitric acid) was added to 100 mL ammoniated silver nitrate and added to washed slides. Slides were incubated 0.5–6 min, or until turned light brown. Slides washed 1 × 1 min in ammonia water followed by 4x washes distilled water. Slides were then dehydrated in ascending ethanol/xylene and coverslipped with Cytoseal XL. Sections were imaged using brightfield at 20X on the Olympus NanoZoomer 2.0-HT slide scanner (Hamamatsu).

### Tissue homogenization

Frozen human tissue was placed on wet ice until just thawed and then immediately placed in 500 μL of PBS + protease inhibitor (Roche) in a 2 mL glass dounce homogenizer. Tissue was dounce homogenized with 30 up/down strokes on ice by hand. The homogenate was transfer to a 1.5 mL Eppendorf tube and centrifuged at 3,000 × g for 10 min at 4°C. The supernatant was collected and aliquoted so that no sample was frozen/thawed more than three times. A bicinchoninic acid assay (BCA, Thermo Scientific Pierce) was performed to determine total protein concentration.

### SDS-PAGE and western blot

Ten to thirty micrograms of total protein per well was loaded on 4–12% Bis-Tris SDS-PAGE gels (Invitrogen) and run in MES buffer (Invitrogen). Proteins were transferred to Immobilon polyvinylidene fluoride membrane (PVDF) (EMD Millipore) and incubated overnight at 4°C with primary antibodies anti-rabbit polyclonal total tau (1:2,000, DAKO), anti-mouse monoclonal phospho-tau PHF-1 (1:2,000, gift from Peter Davies), anti-chicken GAPDH (1:2,000, Abcam), anti-rabbit NeuN (1:1,000, Cell Signaling), anti-mouse PSD-95 (1:1,000, Abcam), anti-chicken beta-tubulin (1:1,000, Aves Labs), or anti-rabbit actin (1:1,000, Abcam) in 1:1 Odyssey blocking buffer:distilled water. Blots were washed 3x 10 min in Tris buffered saline + 0.25% Tween (TBS-T) the next day, incubated with infrared secondary anti-mouse 800, anti-rabbit 680, or anti-chicken 800 (1:2,000, Licor) antibodies in blocking buffer for 1.5 h at room temperature, washed 3x 10 min in TBS-T, and imaged on an Odyssey Infrared Imaging System (Licor). Blots were converted to grayscale and densitometry analysis was performed in ImageJ (NIH v1.51n). For the synaptosome and cytosolic western blots, the PHF1:Total tau ratio was calculated by taking the densitometry value for PHF1 and dividing it by the total tau densitometry value.

### Enzyme-linked immunosorbent assay (ELISA)

The ELISA was performed using 3,000 × g PBS soluble lysate. Total tau was measured using the Meso Scale Diagnostics (MSD) total human tau ELISA (MSD) and the manufacturer's protocol was followed. Plates were developed using the MESO QuickPlex SQ 120 Plate Reader (MSD). Samples were run in triplicate and fit to an eight-point standard curve for total tau concentration determination.

### *In vitro* tau seeding activity

*In vitro* tau seeding activity was assessed as previously described (Holmes et al., 2014; Furman et al., 2015). HEK293 cells stably expressing the repeat domain of Tau with the P301S mutation (Tau-RD^P301S^) fused with cyan fluorescent protein (CFP) and yellow fluorescent protein (YFP) were plated in poly-D-lysine treated 96-well clear bottom plates (Corning) at 30,000 cells/well. The following day when cells were at 60–70% confluency, cells were transduced with 1.0 μg/well lysate plus 1% Lipofectamine 2000 (Invitrogen) diluted in OptiMEM (Thermo Fisher). For the total tau normalization seeding study, 50 μL of 40 ng/mL total tau was used. Each lysate was applied in triplicate. Cells were exposed to lysate for 20–24 h. At the time of collection, cells were trypsinized and transferred to 96-well U-bottom plates (Corning) using chilled DMEM + 10% fetal bovine serum (FBS) to inhibit the activity of the trypsin. Cells were pelleted at 1,000 xg, resuspended in freshly-made 2% paraformaldehyde (PFA) (Electron Microscopy Services) for 10 min at room temperature in the dark, and pelleted at 1,000 × g. Cells were resuspended in chilled PBS and run on the MACSQuant VYB (Miltenyi) flow cytometer. CFP and Forster resonance energy transfer (FRET) were both measured by exciting the cells using the 405 nm laser and reading fluorescence emission at the 405/50 and 525/50 nm filters, respectively. To quantify the FRET signal, a bivariate plot of FRET vs. the CFP donor was generated and cells that received lipofectamine alone were used to identify the FRET-negative population. Using this gate, the “tau seeding” value was calculated by multiplying the percent of FRET-positive cells by the median fluorescence intensity of that FRET-positive population. 40,000 cells per well were analyzed. Data was analyzed using the MACSQuantify software (Miltenyi). Four carry-over samples were used for each plate to normalize across multiple plates run at the same time. Immediately prior to collection, representative images of HEK293 cells with aggregates were taken using the ZOE Fluorescent Cell Imager (BioRad) using the green fluorescent protein (GFP) channel.

### Synaptosome and cytosol preparation

The Synaptosome (SYN) and cytosolic (CYT) fractions were prepared as previously described (Tai et al., 2012; Jhou and Tai, 2017), with minor modifications. See Supplementary Figure 1 for the protocol schematic and conditions tested. 200 mg of frozen human brain was gently homogenized with 25 strokes on ice in a Potter-Elvehjem homogenizer in chilled 500 μL of Buffer A (25 mM HEPES pH7.5, 120 mM NaCl, 5 mM KCl, 1 mM MgCl_2_, 2 mM CaCl_2_) supplemented with 1 mM DTT and 1X protease inhibitor (Roche) in a 2 mL glass dounce homogenizer. The homogenate plus 600 μL Buffer A was then passed through 2 layers of Millipore Nylon 80 μm filters (#NY8002500) using 25 mm Filter Holders (Pall Corp, #4320) to remove tissue debris. Seventy microliters of the crude filtered homogenate was aliquoted into pre-TOT labeled tubes and set aside. The saved pre-TOT aliquot was mixed with 70 μL dH20 and 23 μL PBS and passed through a 27 ½ gauge needle three times to prepare the total extract.

To prepare filtered CYT and SYN fractions, the remaining crude 80 μm filtered homogenate was passed through 5 μm Supor membrane filters (Pall Corp, #4650) along with 300 μL of Buffer A to filter out large organelles and nuclei; this filtrate was centrifuged at 1,000 × g for 10 min at 4°C and supernatant was transferred into pre-CYT tubes. The pellet was washed once with Buffer A and centrifuged at 1,000 × g for 10 min at 4°C to generate the SYN pellet. The SYN pellet was resuspended in either 70 μL of Buffer B (PBS, 1.5% SDS, and 2 mM DTT) or 70 μL of PBS plus protease inhibitor. Half of the pre-CYT supernatant was clarified as previously described (Tai et al., 2012) via ultra centrifugation at 100,000 × g for 1 h at 4°C to obtain the Ultra-CYT sample. The other half of the pre-CYT supernatant was centrifuged at 10,000 × g for 15 min at 4°C to remove any remaining debris and obtain the CYT samples.

### Statistical analysis

The data herein were analyzed for statistical significance using the graphing program GraphPad Prism 6. Data were analyzed for normal distribution where possible using the Shapiro-Wilks test. For parametric analyses, two-tailed Student *t*-test, One-way ANOVA with Sidak *post-hoc* multiple comparisons analysis, and Two-way repeated measures ANOVA with Sidak *post-hoc* multiple comparisons analysis was performed. For non-parametric analyses, two-tailed Mann-Whitney and Kruskal-Wallis with Dunnett *post-hoc* multiple comparisons analysis was performed. For correlation analysis, Spearman correlation was performed and the line-of-best fit generated by linear regression analysis. Individual data points represent individual human cases. Data presented as Mean ± standard error of the mean (SEM).

## Results

### Brain regions with rare tau pathology exhibit high levels of tau seeding

**Figure 1:**
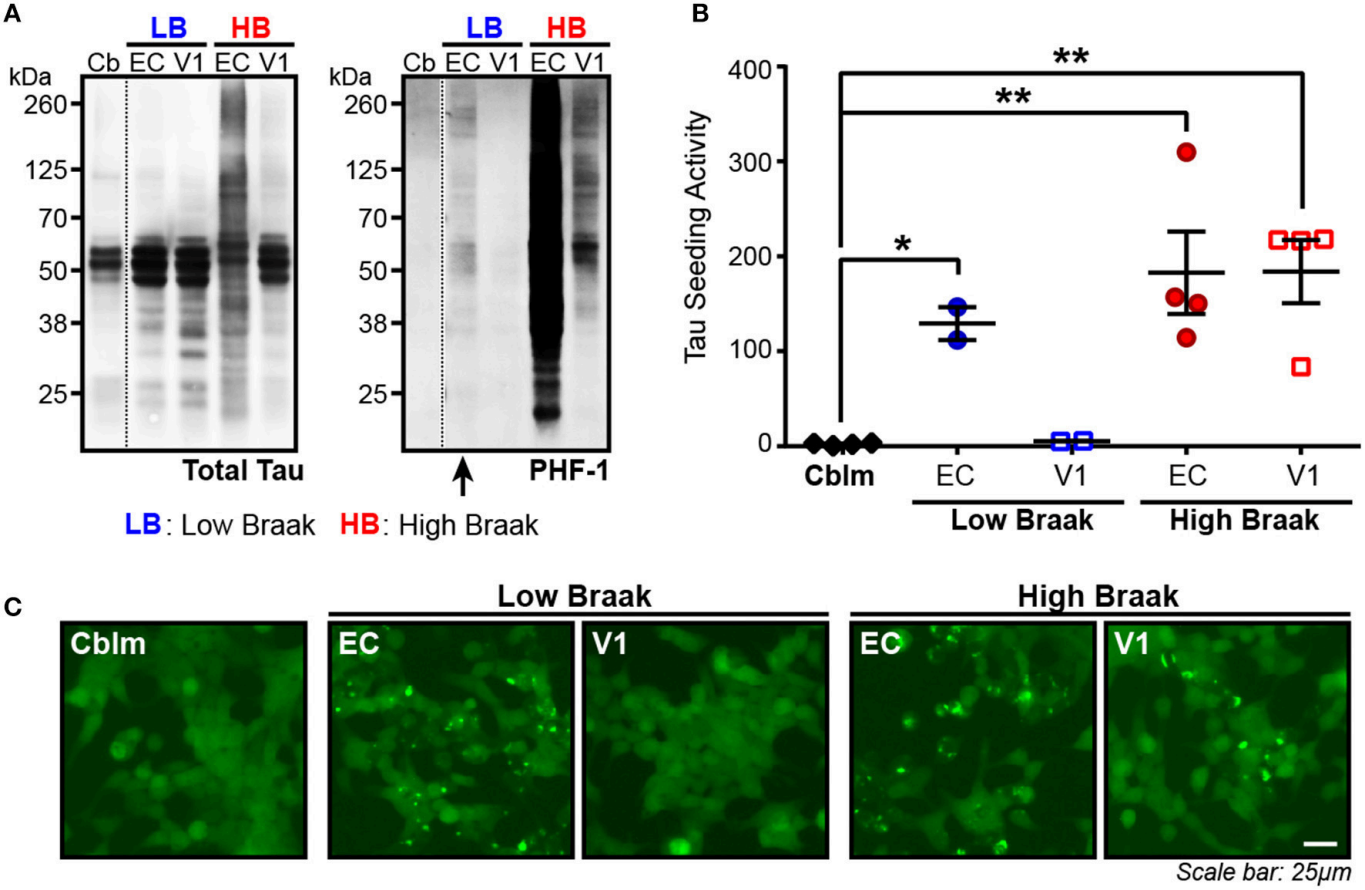
Tau seeding activity can be found in brain regions with rare tau pathology. **(A)** Representative western blot for total tau and PHF-1 phospho-tau of the cerebellum (Cb), entorhinal cortex (EC), and primary visual cortex (V1) of Braak cases with rare NFTs in the EC (Low Braak; LB) and Braak cases with extensive NFTs across the brain (High Braak; HB). Dashed black line indicates discontinuous lanes on the blot. **(B)** Tau seeding as measured on the flow cytometer in the HEK239-Tau^RD^-CFP/YFP seeding cells can be detected in the EC of Low Braak cases despite there being only rare tau pathology. **(C)** Representative photos of induced tau aggregates in the HEK239-Tau^RD^-CFP/YFP cells. One-way ANOVA, Sidak *post-hoc* multiple comparisons. *p < 0.05, **p < 0.01. Individual dots represent individual human cases. Mean ± SEM.

The presence of tau seeding as measured using the tau Tau^RD^-P301S-CFP/YFP *in vitro* biosensor (Holmes et al., 2014) has previously shown an association with the presence of tau pathology, both in tau transgenic mice (Holmes et al., 2014; DeVos et al., 2017) and in human brains (Furman et al., 2017; Nicholls et al., 2017; Nobuhara et al., 2017). Interestingly, tau seeding has also been reported in tissues—both in mice and in human brain lysate—where overt tau pathology has not yet developed or is rare (Holmes et al., 2014; Furman et al., 2017), suggesting that some amount of tau aggregates exist in a brain region before they accumulate to a point of filling the neuronal somatodendritic compartment and are abundantly detected by tau IHC. We analyzed the entorhinal cortex (EC) (first region to be impacted by tau pathology) and the primary visual cortex (V1) (last region to be impacted by tau pathology) in cases with rare NFTs in the EC (Low Braak) and cases with extensive NFTs throughout all regions along the Braak Tau Pathway, including EC and V1 (High Braak). Using the cerebellar vermis from the High Braak brains as an unaffected control region, samples were run on a western blot for phosphorylated tau (antibody PHF-1; epitope 396/404), since the presence of hyperphosphorylated tau has previously indicated the presence of tau seeding (Holmes et al., 2014; DeVos et al., 2017; Nobuhara et al., 2017). Predictably in a High Braak brain, phospho-tau could be detected in both the EC and V1 (Figure 1A). In a Low Braak brain, while there is only rare tau NFTs in the EC observed by IHC, phospho-tau could still be detected in the EC, though not in the V1, perhaps reflecting aberrant tau phosphorylation prior to the development of mild tau pathology at the site of AD tau pathology initiation (Figure 1A). When applied to the tau seeding assay, the EC lysate from the Low Braak cases showed a significant increase in tau seeding activity with no seeding being detected in the V1 Low Braak lysate (Figures 1B,C). Both the EC and V1 lysates from the High Braak cases showed significant tau seeding. While the presence of PHF-1 tau here predicted the presence of seeding, it did not correlate with the amount of seeding activity.

To then test whether the lack of seeding in the cerebellar samples was indeed due to a lack of aggregated tau and not due to less total tau protein, we measured total human tau levels in the cerebellar and EC samples of Braak VI cases and added equal tau amounts to the seeding assay. With this total tau normalization, there was still no detectable seeding activity in the cerebellar samples while all EC samples showed detectable seeding (Supplementary Figure 2). Similar to what has been previously reported, these data show that substantial tau seeding can be detected in both the presence of extensive tau pathology as well as when there are only the rare detectable NFTs.

### Tau seeding is detected in brain regions along the braak tau pathway that are not yet impacted by tau pathology

**Figure 2:**
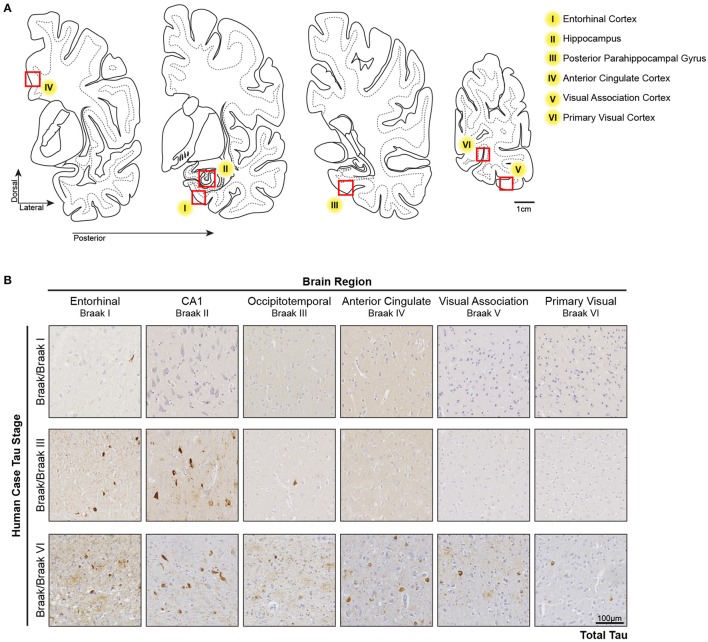
Tau Pathology along the Tau Braak Pathway. **(A)** Brain regions collected from human cases ranging from Braak I–Braak VI (Table 1). Each region corresponds to a region in the Braak Tau Pathway. They are as follows: Braak I, entorhinal cortex; Braak II, hippocampus; Braak III, posterior parahippocampal gyrus; Braak IV, anterior cingulate cortex; Braak V, visual association area; Braak VI, primary visual cortex. Additionally, the cerebellar vermis was collected as an unaffected control region. **(B)** Representative tau IHC photos of tau pathology in brain regions along the Braak Tau Pathway in different Braak staged cases. The Occipitotemporal Gyrus and Posterior Parahippocampal Gyrus can both be use for Braak III staging.

In the Low Braak cases, tau seeding could be detected in the EC, but not the V1 (Figure 1). Since tau pathology consistently begins in the EC in human AD brains, we hypothesized that tau seeding in the EC of Low Braak patients is a predictor of further developing tau pathology. To explore this hypothesis, we collected a series of tissues along the Braak Tau Pathway in a series of human cases (Table 1) ranging from Braak I to Braak VI (Figure 2). Each region collected represents an area that tau pathology would be found in the staging of human brains for Braak status. The regions and corresponding Braak stage are as follows: Braak I, EC; Braak II, Hippocampus (Hp); Braak III, Posterior Parahippocampal Gyrus (PPG); Braak IV, Anterior Cingulate Cortex (AC); Braak V, Visual Association Cortex (VA); Braak VI, Primary Visual Cortex (V1). Brains were staged based on the furthest region tau pathology had reached; a Braak IV brain had at least mild tau deposition in only the EC, Hp, PPG, and AC. The cerebellar vermis was also collected from all brains as an unaffected control brain region.

**Figure 3:**
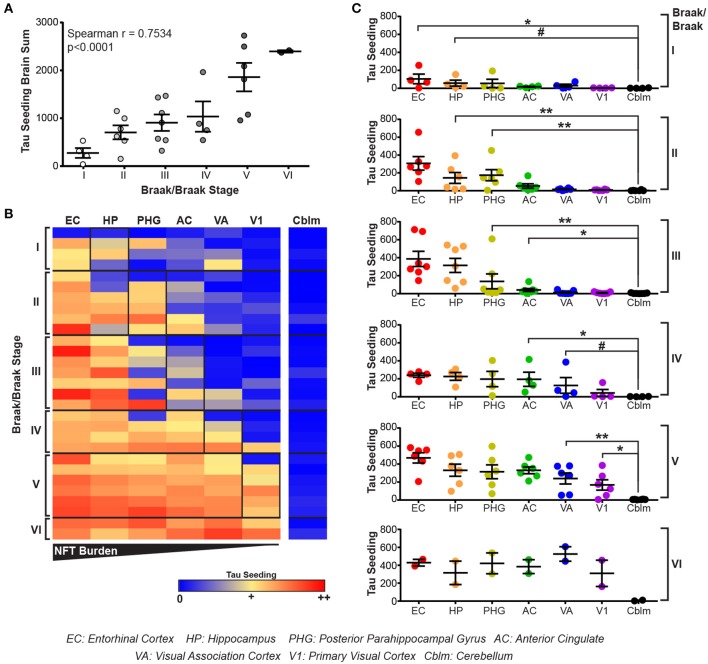
Tau seeding activity precedes tau pathology along the Braak Tau Pathway. **(A)** Lysates along the Braak Tau Pathway for cases ranging from Braak I to Braak VI were all run on the tau seeding assay. Tau seeding values across the all brain regions analyzed were aggregated into a single value for each case and plotted against the Braak status of that brain. A significant direct correlation emerged, whereby as the Braak stage of the brain increases, so does the tau seeding activity across the whole brain. Spearman correlation. **(B)** Heatmap of tau seeding across the brain regions in the Braak Tau Pathway in human brains ranging from Braak I to Braak VI. Regions are organized left to right according to the progression of tau pathology throughout the course of AD; the EC is the first region to be impacted by tau pathology and the V1 is the last region. **(C)** Plots broken up based on the Braak stage of the brains. When compared to the unaffected cerebellum, tau seeding can be detected at least one region past where tau pathology is detected. Kruskal-Wallis test, Dunn's *post-hoc* multiple comparisons. #*p* < 0.01, *p < 0.05, **p < 0.01. Individual dots represent individual human cases. Mean ± SEM.

All brain regions from the human cases were dounce homogenized in 1X PBS and the supernatant from a 3,000 × g spin collected. This lysate was then applied to the tau seeding assay to measure tau seeding along the Braak Tau Pathway. As an initial assessment of tau seeding across the different tau Braak stages, tau seeding across all brain regions for each case was aggregated into a single “total brain” seeding value and plotted against the Braak stage for that brain (Figure 3A). A significant direct correlation between tau seeding and Braak stage emerged, similar to what others have seen in human tissue (Furman et al., 2017).

The main advantage to collecting all regions of the Braak pathway, however, lies in being able to look one region past where tau pathology has been identified. When tau seeding across all regions of all cases is plotted, it becomes evident that tau seeding is detectable in brain regions were tau pathology has not yet reached (Figure 3B). This tau seeding often precedes tau pathology by one region along the Braak Tau Pathway (Figure 3C). While the group mean results are in accordance with previous reports that tau seeding can be detected in brain regions that, according to their tau Braak status, do not yet have overt tau pathology (Furman et al., 2017), the patient to patient variability is worth noting. Even within a single tau Braak stage, not all cases follow the same tau seeding pattern, highlighting the complex heterogeneity of Alzheimer's disease. Even with this variability, these data cumulatively suggest that tau aggregates develop within a defined brain region and, while accumulating over time to a point of being detectable by IHC, simultaneously traverse along axons to the next synaptically connected region along the Braak Tau Pathway.

### Brain regions consisting of almost exclusively neuronal axons exhibit tau seeding activity

**Figure 4:**
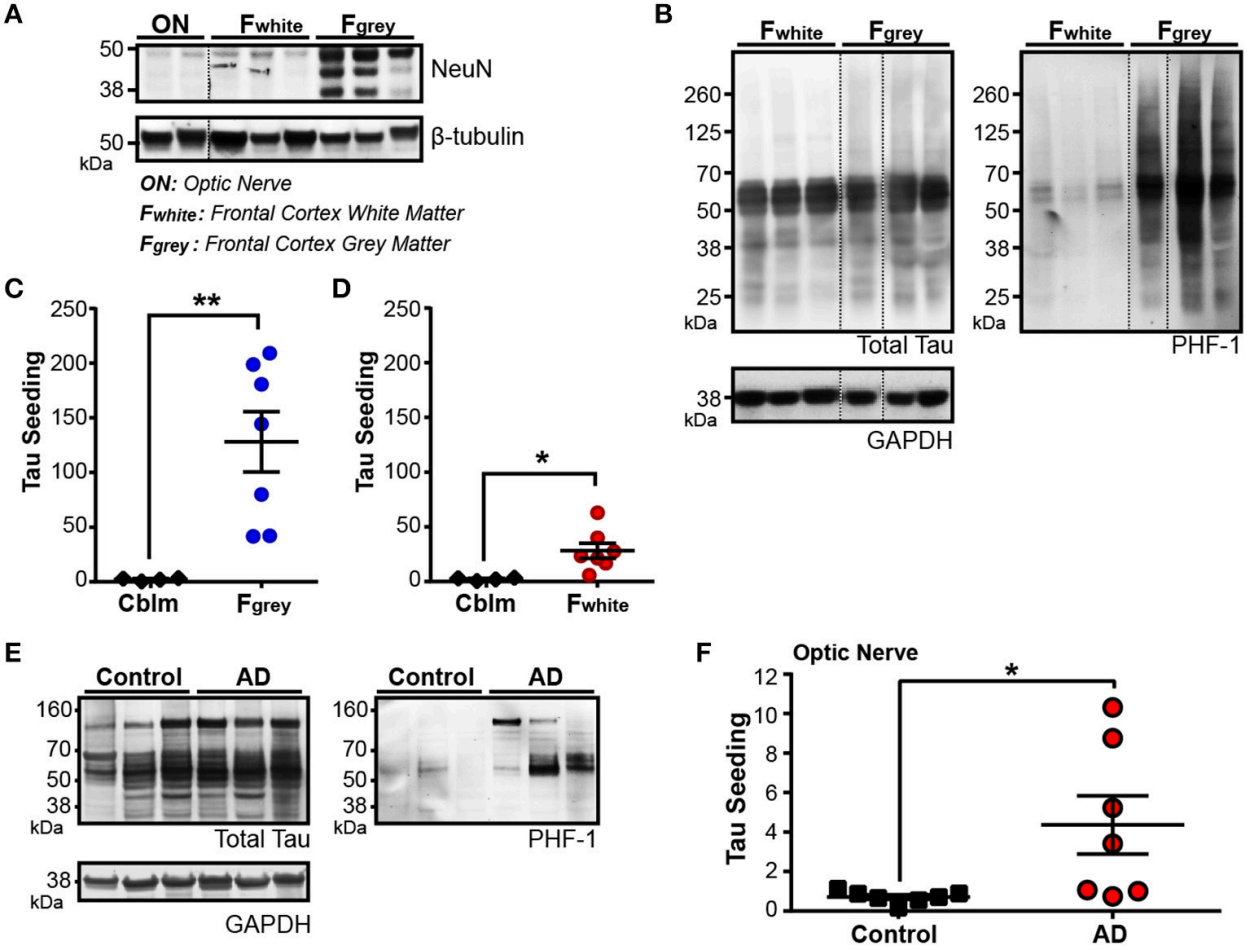
Tau seeding activity is found in axonally-rich brain regions with few to no neuronal cell bodies. **(A)** Representative western blot for pan-neuronal nuclei marker NeuN in optic nerve (ON), frontal cortex white matter (F_white_), and frontal cortex gray matter (F_gray_) from Braak V/VI human AD cases. Dashed black line indicates discontinuous lanes on the blot. **(B)** Western blot of total and PHF1 phospho-tau in F_white_ and F_gray_ matter samples, demonstrating that a small amount of phospho-tau exists in the F_white_ matter, despite there being almost no neuronal cell bodies. **(C,D)** Tau seeding is detected in both the F_gray_ lysates **(C)** and corresponding F_white_ lysates **(D)**. The F_gray_ and F_white_ matter samples were run at the same time on the seeding assay. **(E)** Western Blot of total and PHF1 phospho-tau in control and Braak V/VI AD optic nerves. Phopsho-tau is more readily detected in AD optic nerves. **(F)** Tau seeding can be registered in optic nerve samples from AD human cases, despite there being no neuronal cell bodies. Two-tailed student's *t*-test. *p < 0.05, **p < 0.01. Individual dots represent individual human cases. Mean ± SEM.

For seed competent tau aggregates to move along the Braak Tau Pathway transneuronally, they must at some point exist in the axonal compartment of the neuron. To test this idea, we collected two regions of the human brain that are enriched for neuronal axons and lack neuronal cell bodies: the frontal white matter tracts (F_white_) and the optic nerve (ON). A decrease in NeuN—a pan-neuronal marker that is expressed almost exclusively in the neuronal cell body and nucleus—is seen in both the ON and the F_white_ lysates as compared to the neuronal cell body rich adjacent Frontal gray matter (F_gray_) (Figure 4A). To ensure overt tau pathology was in the F_gray_ matter, Braak V/VI cases were chosen. High levels of phospho-tau were detected in the F_gray_ matter and, interestingly, a small amount of phospho-tau could also be identified in the F_white_ matter despite there being very few neuronal cell bodies and no NFTs in the white matter tracts (Figure 4B). The F_gray_ matter was run on the tau seeding assay and indeed, tau seeding was detected in all cases (Figure 4C). To then test whether the F_white_ matter also contained seed competent tau despite being relatively devoid of neuronal cell bodies, the F_white_ lysate was applied to the same seeding assay and a significant amount of tau seeding was detectable (Figure 4D). It is interesting to point out that while the total tau levels between the F_gray_ and F_white_ matter are similar, there is much higher levels of seeding activity in the F_gray_ matter. This could reflect a number of different possibilities, such as the majority of the seed competent tau exists in the somatodendritic region of the neuron and is the source of axonally trafficked aggregated tau or, alternatively, axonal tau can misfold directly in the axon but to a much lesser extent than somatodendritic tau.

While there are significantly less neuronal cell bodies in the F_white_ matter as compared to the F_gray_ matter, up to 1% of the cell bodies in the white matter are neuronal (García-Marín et al., 2010). The ON, however, is a unique part of the human central nervous system in that it is not only completely devoid of neuronal cell bodies, but the axonal projections in the ON derive from the retinal ganglion cells, a neuronal cell type that does not develop NFTs (Blanks et al., 1989; Ho et al., 2014), and synapses onto the lateral geniculate nucleus. Interestingly, in Braak V/VI cases, we found detectable levels of both phospho-tau (Figure 4E) and tau seeding (Figure 4F) in the ONs (but not in Braak 0-II cases), highlighting the notion that seed competent tau aggregates exist in other neuronal compartments outside of the neuronal cell body. It is unclear from this assay if the tau aggregates derive from the RGCs or the synaptically connected LGN. Nonetheless, tau seeding is detectable along the axons in the ON.

### Tau seeding activity is enriched in synapses along the braak tau pathway

**Figure 5:**
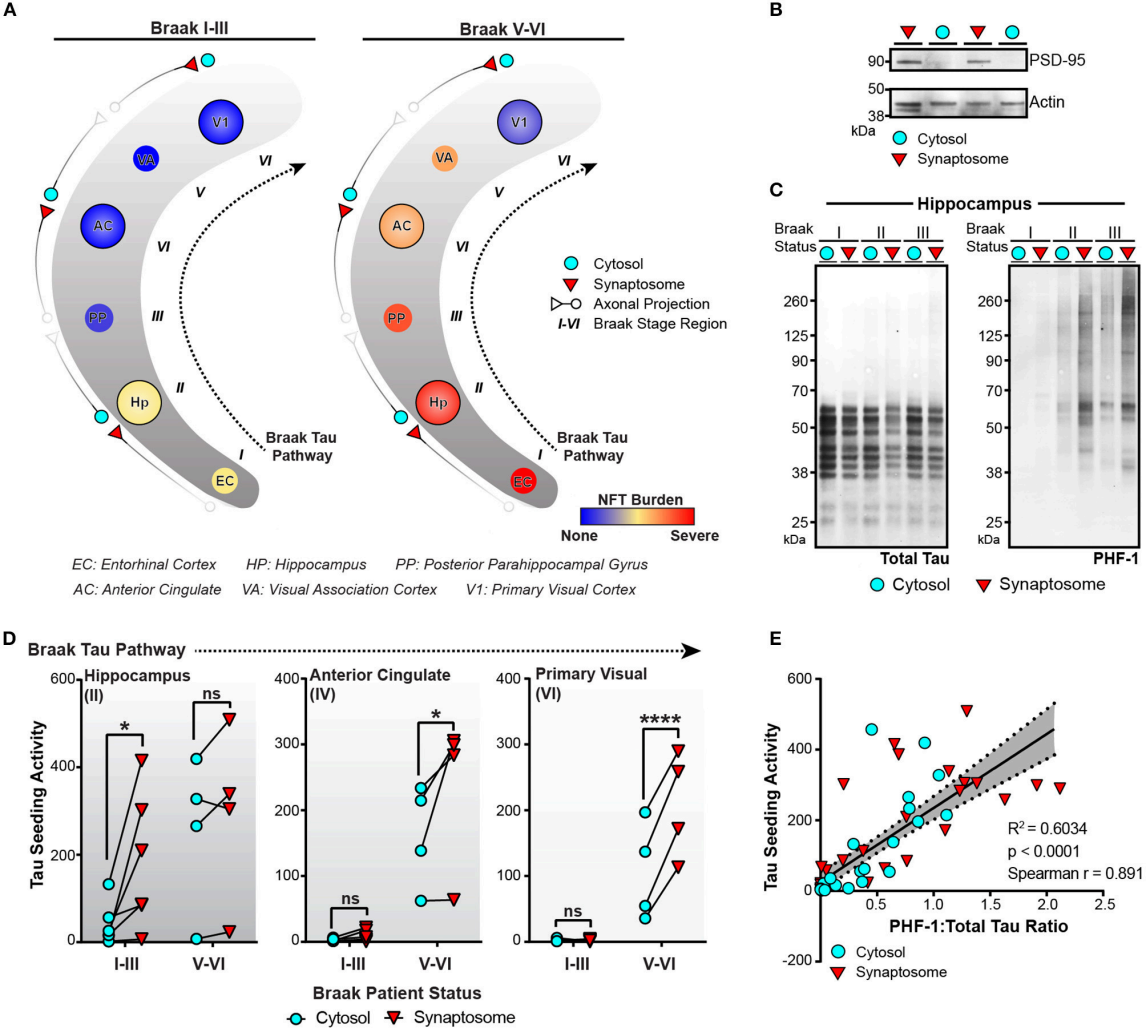
Tau seeding activity is enriched in synapses along the Braak Tau Pathway. **(A)** Schematic of the Braak Tau Pathway. The cytosol and synaptosome fractions were isolated from the Hippocampus (Hp), Anterior Cingulate (AC), and Primary Visual cortex (V1) of human cases. Cases were broken up into early AD, Braak I–III—or late AD, Braak V–VI. The tau neurofibrillary tangle (NFT) burden for each brain region along the pathway shows not only a difference in how far tau pathology has reached, but also the NFT burden level—mild, moderate, or severe— in those regions. **(B)** Representative western blot showing the enrichment of PSD-95 (a synaptic protein) in the isolated synaptosome fraction compared to the cytosolic fraction. Actin was used as a loading control. **(C)** Cytosolic and synaptosome fractions from Braak I–III cases were run on western blot for total tau (left) and PHF1 phospho-tau. **(D)** All cytosolic and synaptosome fractions were run on the tau seeding assay and the cytosolic and synaptic tau seeding levels plotted across the Braak Tau Pathway for both the Braak I–III and Braak V–VI groups. The lines between the cytosolic and synaptic seeding values represent the same case. Two-way repeated measures ANOVA, Sidak *post-hoc* multiple comparisons. **(E)** The ratio of PHF1:Total tau on the western blot for all samples was plotted against the tau seeding levels of all the same samples, yielding a significant direct correlation. Overlaid is the line of best fit and the 95% confidence interval bands determined by linear regression. Spearman correlation. *p < 0.05, ****p < 0.0001. Individual dots represent individual human lysate samples.

Tau seeding can be detected in regions of the human brain devoid of neuronal cell bodies and enriched for axons. For aggregates of tau to propagate transneuronally, one proposed mechanism is the movement of aggregates from one neuron to the next across synapses. We have previously detected an increase in phosphorylated oligomeric tau in the synaptosome fraction of AD brain lysate when compared to control tissue (Tai et al., 2012, 2014; Perez-Nievas et al., 2013). To expand on these results and test whether aggregates of tau can be detected in synapses along the Braak Tau Pathway, the cytosolic and synaptosome fractions were isolated (Supplementary Figure 1) in three different brain regions from early AD (Braak I–III) and late AD (Braak V–VI) cases (Figure 5A). The tau NFT burden reveals mild/moderate levels of NFTs in the first three regions of the Tau Braak Pathway of the Braak I–III group, while the Braak V–VI cases show much more severe NFT burden in the earlier Braak Tau Pathway regions that tapers off in later regions (Figure 5A). Synaptosome and cytosolic fractions were prepped and when run on western blot, the synaptic-specific protein post-synaptic density 95 (PSD-95) was heavily enriched in the synaptic vs. cytosolic fraction (Figure 5B), demonstrating the efficacy of the synaptic enrichment preparation. The cytosolic and synaptic fractions were run on western blot and probed for both total tau and phospho-tau (Figure 5C). While the levels of total tau remained similar across the different cases and fractions, there was a visible increase in the phospho-tau:total tau ratio in the synaptic fractions when compared to the cytosolic fraction from the same case (Figure 5C).

We next ran the cytosolic and synaptic fractions from all cases across all regions on the tau seeding assay to measure the amount of tau seeding activity in the cytosol and synapses along the Braak Tau Pathway in both early and late AD conditions (Figure 5D). In the hippocampus, one of the earlier regions to be impacted by tau pathology, there was a significant enrichment of tau seeding in the synapses as compared to the cytosol in the Braak I–III cases, though interestingly, not in the more heavily impacted Braak V–VI cases. In the later regions in the Braak Tau Pathway—the Anterior Cingulate and Primary Visual Cortex—, there was almost no seeding in the early AD Braak I–III cases. However, in the late AD cases, we observed the same phenomenon as in the early AD hippocampus—a significant increase in the seeding activity in the synapses as compared to the cytosolic fraction. From these data, it suggests that in brain regions with mild to moderate tau pathology, there is an enrichment in phosphorylated seed competent tau species in the synapses, lending support to the idea of tau aggregates moving transneuronally through the human AD brain along synaptically connected brain regions.

## Discussion

In keeping with previous *in vivo* and human literature (Holmes et al., 2014; Furman et al., 2017), we have recapitulated the finding that tau seeding—i.e., the ability of an aggregate of tau to recruit naïve monomeric tau—can be detected before extensive visible tau pathology (Figure 1). In fact, this seed competent tau precedes tau pathology along the Braak Tau Pathway by at least one region (Figures 2, 3). Further, tau seeding can be detected in two axonally rich regions of the human brain that are devoid of neuronal cell bodies (frontal white matter tracts and optic nerve), suggesting that aggregates of tau can move along axons as a possible mode of transmission through the brain (Figure 4), a mechanism that has been shown to be feasible *in vitro* (Wu et al., 2013; Takeda et al., 2015). Finally, we demonstrated in regions of the human AD brain that are not yet substantially impacted by tau pathology that tau seeding is significantly enriched in the synapses as compared to the cytosol (Figure 5), further providing evidence that tau aggregates can move along synaptically connected brain regions as Alzheimer's disease progresses through the human brain.

It has long been known that tau pathology appears in human AD brains in a very spatially consistent manner, starting in the entorhinal cortex and ultimately culminating in the primary visual cortex. These pathology data have laid the framework for the theory that tau aggregates move through the brain transneuronally along this pathologically defined Braak Tau Pathway, ultimately resulting in overt tau pathology and neuronal cell death along the same pathway. Numerous *in vitro* studies have demonstrated cellular uptake of tau aggregates, the ability of tau aggregates to recruit and template naïve monomeric tau, and the fact that tau aggregates can be released from neurons (Frost et al., 2009; Kfoury et al., 2012; Holmes et al., 2013; Wu et al., 2013; Sanders et al., 2014; Takeda et al., 2015; Nobuhara et al., 2017). Further, tau aggregates can be seen moving both anterograde and retrograde along axons (Wu et al., 2013). Expounding on these *in vitro* studies, several groups have now reported that this same phenomenon can occur *in vivo*, both in genetic models that drive tau expression in the entorhinal cortex (de Calignon et al., 2012; Liu et al., 2012), as well as injection models that show both the recruitment of endogenous tau and spread of aggregates to synaptically connected brain regions (Clavaguera et al., 2009, 2013; Iba et al., 2013; Ahmed et al., 2014; Dujardin et al., 2014; Sanders et al., 2014; Stancu et al., 2015; Narasimhan et al., 2017). These results set the stage for investigating the movement of tau aggregates through the human brain along the pathologically defined Braak Tau Pathway. It was recently demonstrated that tau seeding can be detected in the human AD brain before the onset of tau pathology (Furman et al., 2017), a phenomenon that was previously seen in tau transgenic mice (Holmes et al., 2014). Our results herein recapitulate these data by identifying seed competent tau in brain regions along the Braak Tau Pathway that do not yet have overt tau IHC pathology. We further analyzed axonally rich regions of the human brain almost or completely devoid of neuronal cell bodies—frontal white matter and optic nerve, respectively—and observed seed competent tau existing outside of the neuronal soma compartment. These regions of the human brain are densely comprised of axons, though it should be noted that they also contain glia cells, including oligodendrocytes, astrocytes, and microglia. Microglia have been shown *in vivo* to take up tau aggregates (Bolós et al., 2015; Luo et al., 2015), though in AD white matter, we do not see any pathological tau deposition. Glial cells also have significantly less tau expression than neurons (Leyns and Holtzman, 2017) and thus, if they contain detectable tau aggregates and seeding activity, the aggregates are likely coming from neighboring neuronal axon projections where tau is much more abundant.

One proposed method of tau aggregate transneuronal spread is the synaptic release of tau aggregates that then get taken up by the adjoined neuron. We and others have previously shown that phosphorylated-tau oligomers are enriched in the synapses of human AD brain tissue, as compared to control, while total tau remains the same between the synaptic and cytosolic fractions (Henkins et al., 2012; Tai et al., 2012, 2014; Perez-Nievas et al., 2013; Sokolow et al., 2015; Zhou et al., 2017). In these studies, oligomeric tau could be detected in both the pre-synaptic and post-synaptic particles as assessed by IHC (Tai et al., 2014), though the synaptosome preparation used in these past studies and our current work herein isolates both pre- and post-synapses together. The isolation of both pre- and post-synapses together, while an important first step, invites futures studies looking at the seeding levels specifically in pre- and post-synaptic fractions. This would allow for the further dissection of whether tau aggregates can exist not only in post-synapses as is predicted with the somatodendritic localization of tau pathology, but also in the pre-synaptic compartment which would further suggest a trans-synaptic mechanism of propagation.

In addition to looking at both pre- and post-synapses together, previous studies looking at tau oligomers in synaptic fractions were generated using only one region. Our results using synaptic and cytosolic fractions along three regions of the Braak Tau Pathway not only recapitulated the increased ratio in pTau:Total tau in the synaptic fractions, but also demonstrated for the first time a significant increase in tau seeding at the synapse in human brain tissue. Interestingly, we noted that in the presence of severe NFTs, there was not a significant enrichment of tau seeding in synapses, unlike regions with mild-moderate NFT burden. This could be due to a number of reasons. In brain regions heavily impacted by NFTs, there is a substantial increase in cytosolic tau accumulation as seen by tau pathology staining, reflecting the re-organization of tau to the soma. This reorganization would likely increase the amounts of cytosolic tau seeds and result in more similar tau seeding value between cytosol and synapse fractions. There is also synaptic loss that corresponds to the presence and level of tau pathology (Scheff et al., 2007; Yoshiyama et al., 2007; Polydoro et al., 2014; Spires-Jones and Hyman, 2014; Pickett et al., 2017), such that tau aggregates may not be able to spread via synaptic connections to the same degree if there are less overall synapses. On the converse, in those regions with only mild to moderate NFT burden that have not yet experienced the same level of cytosolic tau pathology or synapse loss, tau aggregates can still propagate across synapses (Calafate et al., 2015), thus resulting in the local synaptic enrichment of tau seeding. In the rTgTauEC propagation mouse model (de Calignon et al., 2012; Liu et al., 2012), this same phenomenon was observed, whereby human tau spread from the EC to the synaptically connected dentate gyrus early in the course of disease (Pickett et al., 2017). Importantly, this spread of human tau occurred before the advent of synapse and neuronal loss, suggesting that the spread of tau between adjoining neurons is not simply due to the degeneration of axon terminals, but instead is an active early event in the disease progression.

The data generated here and in previous literature looking at tau seeding levels in the human brain have been done in a cross-sectional manner from frozen autopsy tissue. Tracking tau aggregates traversing from one brain region to another in a living person is not yet feasible. However, while more crude, the presence of tau NFTs can be analyzed longitudinally in the living human brain using tau positron emission tomography (PET) imaging (Newberg et al., 2002). When coupled with Amyloid-beta (Aβ) Pittsburg compound B (PiB) PET (Klunk et al., 2004) and fluorodeoxyglucose (FDG)-PET imaging (Newberg et al., 2002), a picture of the spatial and temporal development of tau and amyloid pathologies emerges, helping illuminate how each pathology relates to cell loss as the disease progresses. In living human AD patients, it has now been demonstrated that the spatial and temporal retention of the Tau PET ligand corresponds well with Braak staging of NFT tau pathology (Ossenkoppele et al., 2016; Schöll et al., 2016; Hoenig et al., 2018). When the second major AD pathology—Aβ plaques—is measured in the same patients, the deposition of Aβ and tau as measured by PET show distinct patterns throughout the AD brain (Sepulcre et al., 2016) with tau pathology showing a more direct correlation with neural network functional decline, cortical atrophy, and cognitive decline (Ishiki et al., 2015; Ossenkoppele et al., 2016; Schöll et al., 2016; Hoenig et al., 2018). These PET data not only reaffirm what the field has long seen with tau IHC, but also set the stage to monitor the progression of tau pathology along the Braak Tau Pathway as new tau-based therapeutics are brought to the human clinic.

It should be noted that the biosensor cell line used throughout this study, while having been previously characterized, is an artificial *in vitro* system with the repeat domain of tau containing the P301S point mutation (Holmes et al., 2014; Furman et al., 2015, 2017). In our studies here, we used the cells in conjunction with a lipofectamine treatment purely as a tool to better understand the total amount of seed-competent tau aggregates in the samples tested. Studies have shown that AD derived tau can induce seeding in this assay despite the P301S mutation (Holmes et al., 2014; Furman et al., 2017). Further, lipofectamine was used in our studies as we wanted to know the full extent of seed competent tau in the lysates and to avoid the additional variable of cellular uptake. Importantly, while this study helps demonstrate that tau seeding activity can exist in non-pathology containing regions along the pre-defined Braak Tau Pathway, it should also be noted that the seeding data herein do not provide information as to the potency of each tau aggregate—e.g. how efficient an aggregate of tau can be taken up by a cell and how much seeding each tau aggregate can induce. Future studies that look at treating neurons without lipofectamine and/or normalizing to total or phosphorylated levels of tau will help get at some of these other questions surrounding tau seeding activity from human AD brain lysate.

Knowing that the presence of NFTs is in direct correlation with neuronal death and that tau aggregates appear to propagate through the brain transneuronally, several tau directed therapeutics have emerged for the treatment of AD. In keeping with the idea that tau aggregates move across synapses, one possible treatment is tau immunotherapy—as tau aggregates are released from a neuron, the aggregates can be captured by an antibody and cleared before they can be taken up by the adjoining neuron. This therapy has been shown both *in vitro* and *in vivo* to be successful at preventing both tau pathology formation and propagation (Boimel et al., 2010; Bi et al., 2011; Yanamandra et al., 2013; Castillo-Carranza et al., 2014; Ittner et al., 2015; Nicholls et al., 2017; Nobuhara et al., 2017). Based on these successful studies, tau immunotherapy has been taken to the human clinic by multiple groups for those with early AD (NCT02880956, NCT03289143, NCT03056729). Interestingly, not all epitopes of tau work as well at clearing the seed competent portion of tau pathology (Yanamandra et al., 2013; Nobuhara et al., 2017). To design a tau antibody that has the best chance of preventing pathological tau propagation, the data herein suggest that screening tau antibodies against the synaptic seed relevant tau species may help generate a tau immunotherapy that is unique to the species of tau responsible for AD progression. Other therapies, such as preventing the subsequent uptake (Holmes et al., 2014) or release of tau aggregates, could, based on these data, help slow or prevent the spread of tau aggregates and thus clinical progression of human AD.

## Ethics statement

Human brain tissue was obtained from the Massachusetts Alzheimer's Disease Research Center Brain Bank. All of the subjects or their next of kin gave informed consent for their brain donation. The Massachusetts General Hospital Institutional Review Board approved the study protocol.

## Author contributions

SD and BH conceived and designed the studies and wrote the manuscript. SD, DO, JG, PD, MF, and BH identified which human brain cases and regions to collect from for the Braak staging experiments. The following authors carried out experiments and collected data: SD, BC, RB, CN, DO, JG, AC, CC, and PD. Once the experiments were completed and data collected SD, BC, RB, CN, CC, DO, and BH analyzed and interpreted the results. SD performed the statistical analyses and SD and BH secured funding for the project.

### Conflict of interest statement

The authors declare that the research was conducted in the absence of any commercial or financial relationships that could be construed as a potential conflict of interest.

## Supplementary material

Supplementary Figure 1 Synaptosome protocol outline. **(A)** Schematic of synaptosome prep protocol and different conditions tested. **(B)** Total tau and phospho PHF1 tau blots, demonstrating that boiling the preps did not impact the tau banding patterns. **(C)** Total and phospho PHF1 tau blots, demonstrating that ultra-centrifugation of the cytosolic fraction lead to a large decrease in phospho PHF1 tau, while spinning the cytosolic fraction at the same speed as the synaptosome fraction retained phospho PHF1 tau in the sample.

Supplementary Figure 2 Tau seeding normalized to total tau levels. Biosensor cells treated with lysate normalized to 40 ng/mL total tau. Tau seeding was normalized to cells treated with no lysate (NT). After normalizing to total tau, cerebellum (Cblm) lysate from High Braak cases did not induce tau seeding activity while the entorhinal cortex (EC) lysate shows significantly more seeding compared to both NT and Cblm treated cells. One-way ANOVA, Sidak *post-hoc* multiple comparisons test. ^*^*p* < 0.05. Individual dots represent individual human cases. Mean ± SEM.
