# Supplementary figures and images for: Synaptic Tau Seeding Precedes Tau Pathology in Human Alzheimer's Disease Brain
Source: Front Neurosci. 2018 Apr 24;12:267. doi: 10.3389/fnins.2018.00267 (PMC5928393; doi:10.3389/fnins.2018.00267)

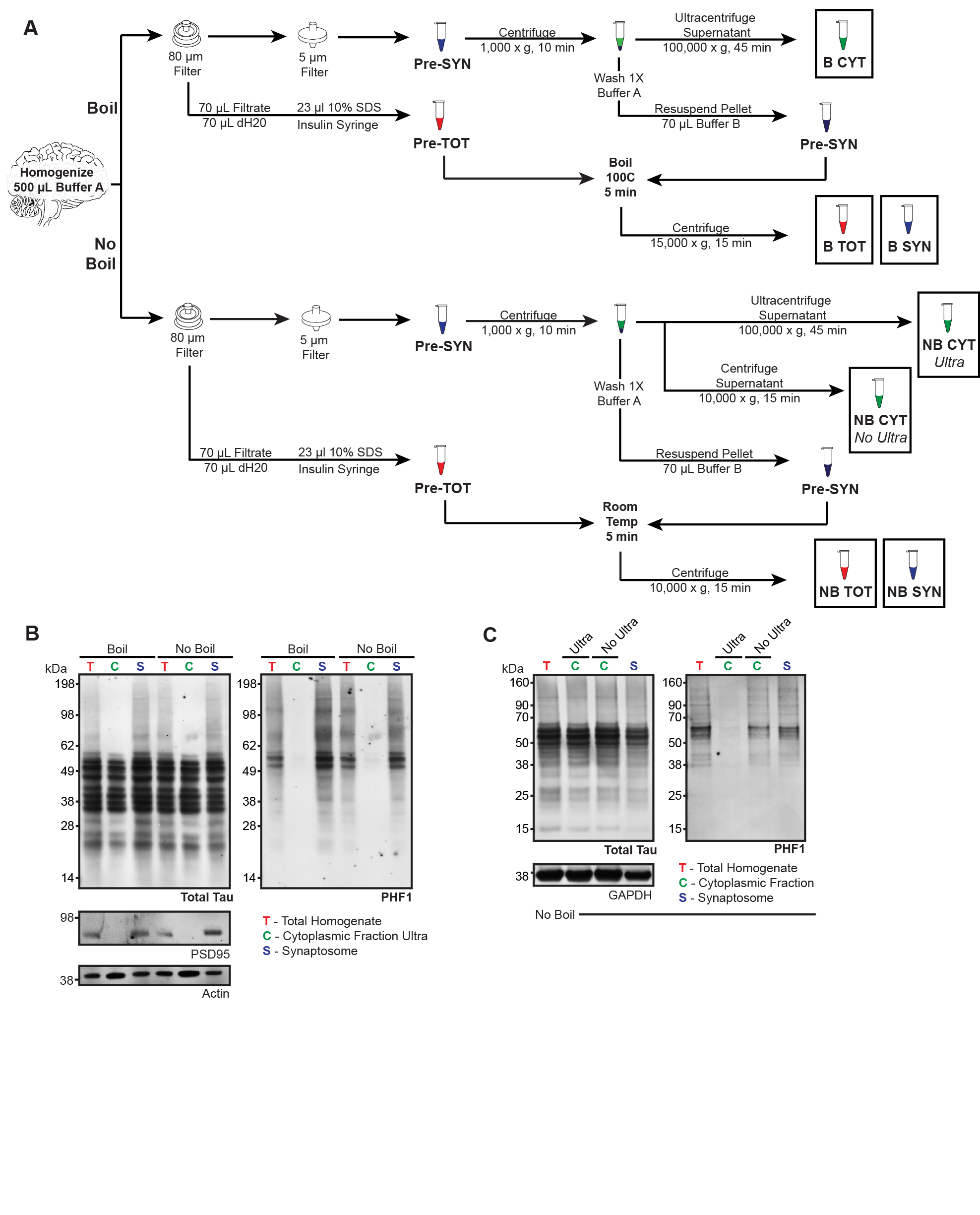

Supplement: Supplementary Figure 1 — Synaptosome protocol outline. (A) Schematic of synaptosome prep protocol and different conditions tested. (B) Total tau and phospho PHF1 tau blots, demonstrating that boiling the preps did not impact the tau banding patterns. (C) Total and phospho PHF1 tau blots, demonstrating that ultra-centrifugation of the cytosolic fraction lead to a large decrease in phospho PHF1 tau, while spinning the cytosolic fraction at the same speed as the synaptosome fraction retained phospho PHF1 tau in the sample. [file Image_1.JPEG]

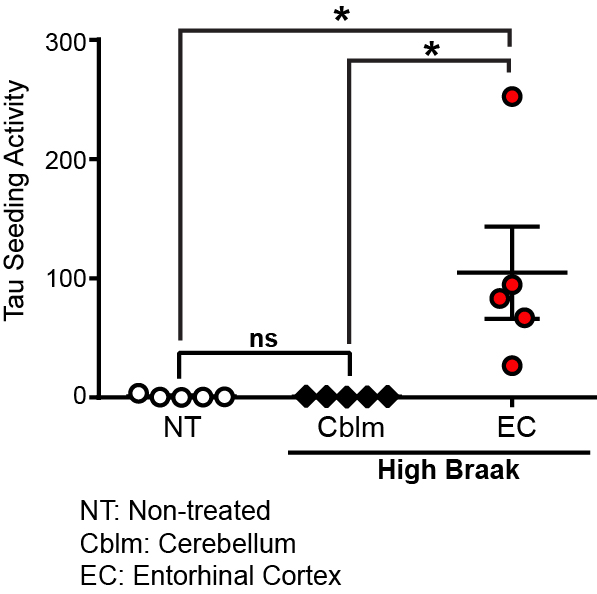

Supplement: Supplementary Figure 2 — Tau seeding normalized to total tau levels. Biosensor cells treated with lysate normalized to 40 ng/mL total tau. Tau seeding was normalized to cells treated with no lysate (NT). After normalizing to total tau, cerebellum (Cblm) lysate from High Braak cases did not induce tau seeding activity while the entorhinal cortex (EC) lysate shows significantly more seeding compared to both NT and Cblm treated cells. One-way ANOVA, Sidak post-hoc multiple comparisons test. *p < 0.05. Individual dots represent individual human cases. Mean ± SEM. [file Image_2.JPEG]
